# Overcoming the bottleneck to widespread testing: a rapid review of nucleic acid testing approaches for COVID-19 detection

**DOI:** 10.1261/rna.076232.120

**Published:** 2020-07

**Authors:** Meagan N. Esbin, Oscar N. Whitney, Shasha Chong, Anna Maurer, Xavier Darzacq, Robert Tjian

**Affiliations:** 1 Department of Molecular and Cell Biology, University of California Berkeley, Berkeley, California 94720, USA; 2 The Howard Hughes Medical Institute, University of California Berkeley, Berkeley, California 94720, USA

**Keywords:** CRISPR, LAMP, RT-PCR, covid-19, sars-cov-2

## Abstract

The current COVID-19 pandemic presents a serious public health crisis, and a better understanding of the scope and spread of the virus would be aided by more widespread testing. Nucleic-acid-based tests currently offer the most sensitive and early detection of COVID-19. However, the “gold standard” test pioneered by the U.S. Centers for Disease Control and Prevention takes several hours to complete and requires extensive human labor, materials such as RNA extraction kits that could become in short supply, and relatively scarce qPCR machines. It is clear that a huge effort needs to be made to scale up current COVID-19 testing by orders of magnitude. There is thus a pressing need to evaluate alternative protocols, reagents, and approaches to allow nucleic-acid testing to continue in the face of these potential shortages. There has been a tremendous explosion in the number of papers written within the first weeks of the pandemic evaluating potential advances, comparable reagents, and alternatives to the “gold-standard” CDC RT-PCR test. Here we present a collection of these recent advances in COVID-19 nucleic acid testing, including both peer-reviewed and preprint articles. Due to the rapid developments during this crisis, we have included as many publications as possible, but many of the cited sources have not yet been peer-reviewed, so we urge researchers to further validate results in their own laboratories. We hope that this review can urgently consolidate and disseminate information to aid researchers in designing and implementing optimized COVID-19 testing protocols to increase the availability, accuracy, and speed of widespread COVID-19 testing.

## OVERVIEW

**FIGURE 1.**
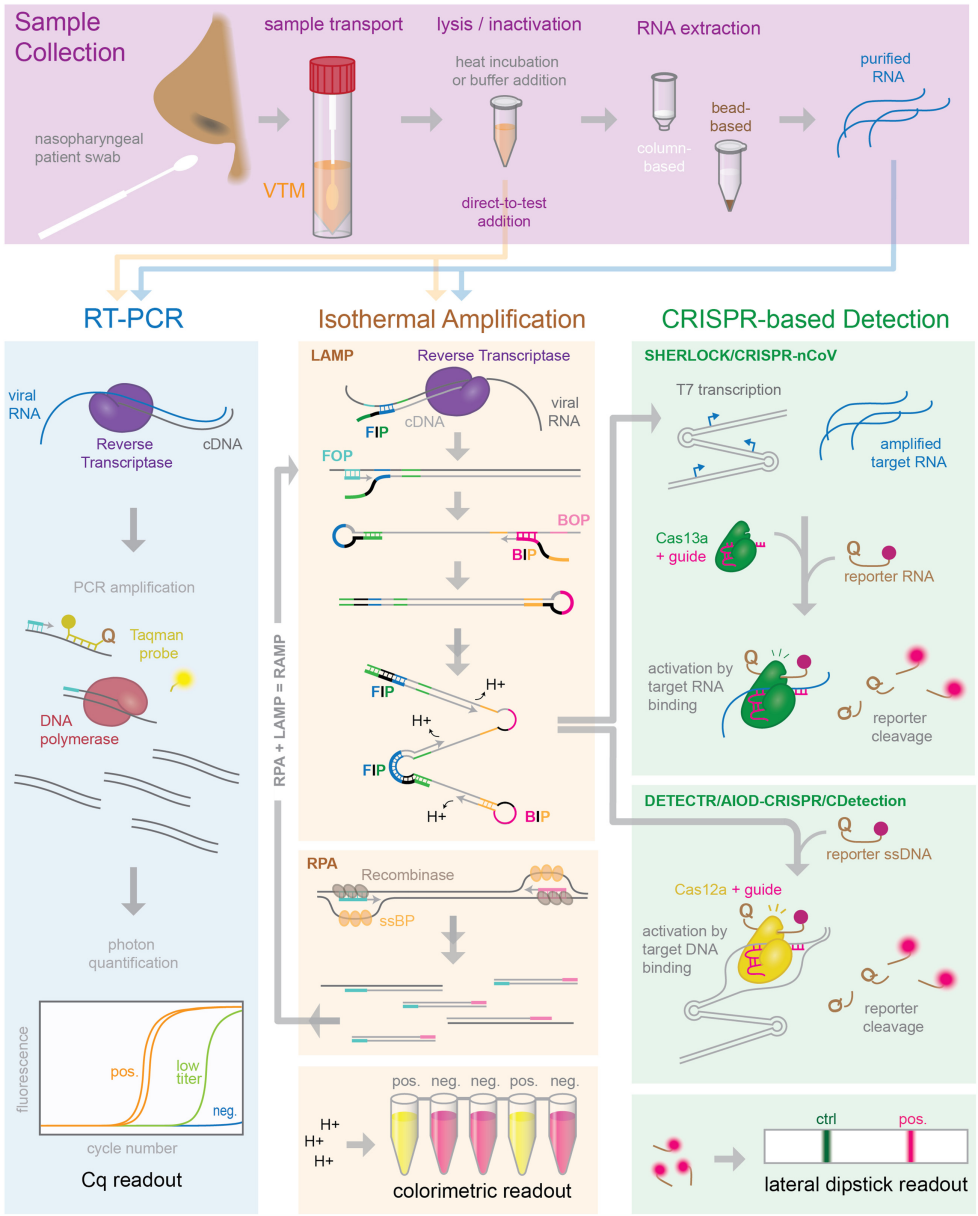
An overview of COVID-19 nucleic acid testing. Samples collected via nasopharyngeal swab are lysed and inactivated, and an amplification reaction is performed using either a crude swab sample or purified RNA. Amplification of specific viral sequences by RT-PCR, LAMP, or RPA is detected using fluorescent or colorimetric dyes, sequence-specific CRISPR-Cas nuclease cleavage of a reporter, or separation of reaction products on a lateral flow dipstick.

On March 11th, 2020, the World Health Organization deemed COVID-19 a global pandemic (World Health Organization 2020). As of April 26th, SARS-CoV-2 infections have been confirmed in almost 3 million people worldwide, yet even this staggering figure is likely to be an underestimate (Elflein 2020). To have any actionable impact on our control of the pandemic propagation, tests should be performed repetitively on a large fraction of the population in order to detect outbreaks before they spread. Current estimates of the testing capacity needed to end the pandemic are in the range of tens of millions of tests per day in the U.S., far above the ∼145,000 tests currently conducted nationally (Goodnough et al. 2020; Irfan 2020). A solution to massively scaling up COVID-19 testing by orders of magnitudes may be aided by an innovative combination of the molecular tools presented here. Current testing approaches fall into two categories—nucleic-acid or serological. Nucleic-acid tests directly probe for the RNA of viruses swabbed from a patient's throat or nasal passage (Fig. 1), while serological tests detect antibodies present in the patient's serum. During the first days of infection, patient viral titers are high and a single patient nasopharyngeal swab may harbor close to 1 million SARS-COV-2 viral particles (Wölfel et al. 2020). However, patient IgG and IgM antibody production, termed seroconversion, typically occurs 5–10 d after the onset of initial symptoms (Wölfel et al. 2020). Therefore, nucleic acid tests offer the earliest and most sensitive detection for the presence of SARS-COV-2 and will be the subject of this review. The RT-PCR test pioneered by the CDC has been deemed the “gold standard” for clinical diagnosis but takes hours to perform and requires specialized reagents, equipment, and training (Centers for Disease Control and Prevention 2020). In the first few weeks of the global SARS-CoV-2 pandemic, required reagents were already in short supply, and researchers and testing centers reported issues acquiring almost every necessary reagent from commercial suppliers—from patient nasopharyngeal swabs to lysis buffer to RNA extraction kits (Akst 2020; Baird 2020). Some testing centers have even been running multiple testing protocols side-by-side to increase throughput and allow for decreased reliance on a single reagent (Soucheray 2020). A few commercial test systems exist but are primarily designed to give single-patient results (Abbott 2020; Xpert Xpress SARS-CoV-2, https://www.fda.gov/media/136314/download). A scalable, high-throughput platform will be required to deliver millions of tests per day. Here we investigate recent advances and approaches to nucleic-acid testing for COVID-19. We highlight some findings from research groups who have compared commercial reagents or created homemade solutions in order to decrease cost or reliance on particular commercial reagents. We also outline several alternative nucleic-acid tests involving isothermal amplification or CRISPR-based detection. Finally, we examine some recent applications of specialized techniques such as sequencing, digital PCR, and DNA nanoswitches as tools for COVID-19 detection. We have tried to be as exhaustive as possible throughout this review, but due to the rapid daily developments in testing we may have unwittingly excluded some published works. Another review by Shen et al. (2020) published in late February may be useful to readers. In this review, we greatly expand the scope to evaluate and compare many more recently published articles, address advancements in sample lysis, direct addition, and novel detection methods, and include a quantitative comparison of these methods including workflow time, cost, and limit of detection.

**FIGURE 2.**
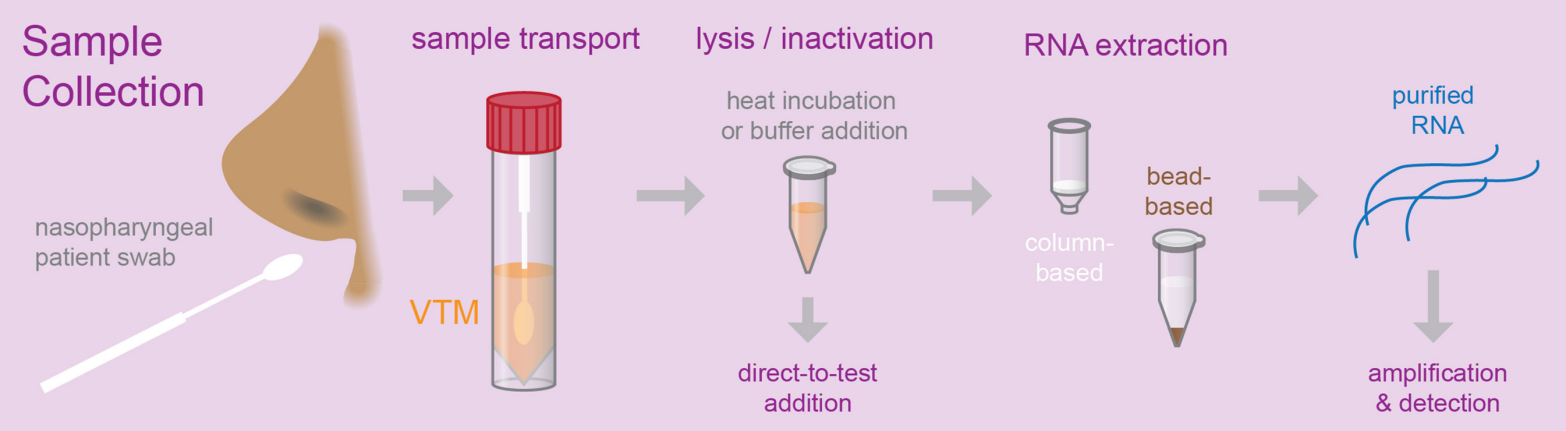
An overview of sample processing. Patient nasopharyngeal swabs are collected and transported for testing. Viral particles are inactivated and lysed by heat and/or lysis buffer addition. Swab sample is then added directly to amplification reactions or RNA is purified from the sample and then amplified.

The general workflow for RT-PCR tests, such as that approved by the CDC, includes three main steps: sample collection and transport, lysis and RNA purification, and amplification (Fig. 2). Typically, a clinician collects a nasopharyngeal swab and transfers it to a vial containing a few milliliters of viral transport medium (VTM), which is transported to a laboratory for testing. Chemical lysis buffers or heating may be used to lyse and inactivate viral particles. The viral RNA is then purified from a fraction of the swab sample (typically 1/20th of the swab) using column-based RNA purification kits or magnetic beads. The eluted purified RNA is then amplified using a one-step master mix containing reverse transcriptase and DNA polymerase enzymes with three primers targeting specific regions of the viral genome. Primers targeting a human gene, such as *RNaseP*, are also included as a positive control for swab collection, RNA extraction and amplification. A spike-in control RNA, such as *MS2* bacteriophage genomic RNA, may alternatively be used. Amplified products can be detected using TaqMan probe fluorescence or DNA-intercalating dyes, and a threshold cycle of amplification is set to distinguish positive from negative results. A test result is typically considered positive if amplification is observed for two or more viral targets, while it is considered negative if amplification is observed for the control RNA but for none of the viral targets (Centers for Disease Control and Prevention 2020).

**FIGURE 3.**
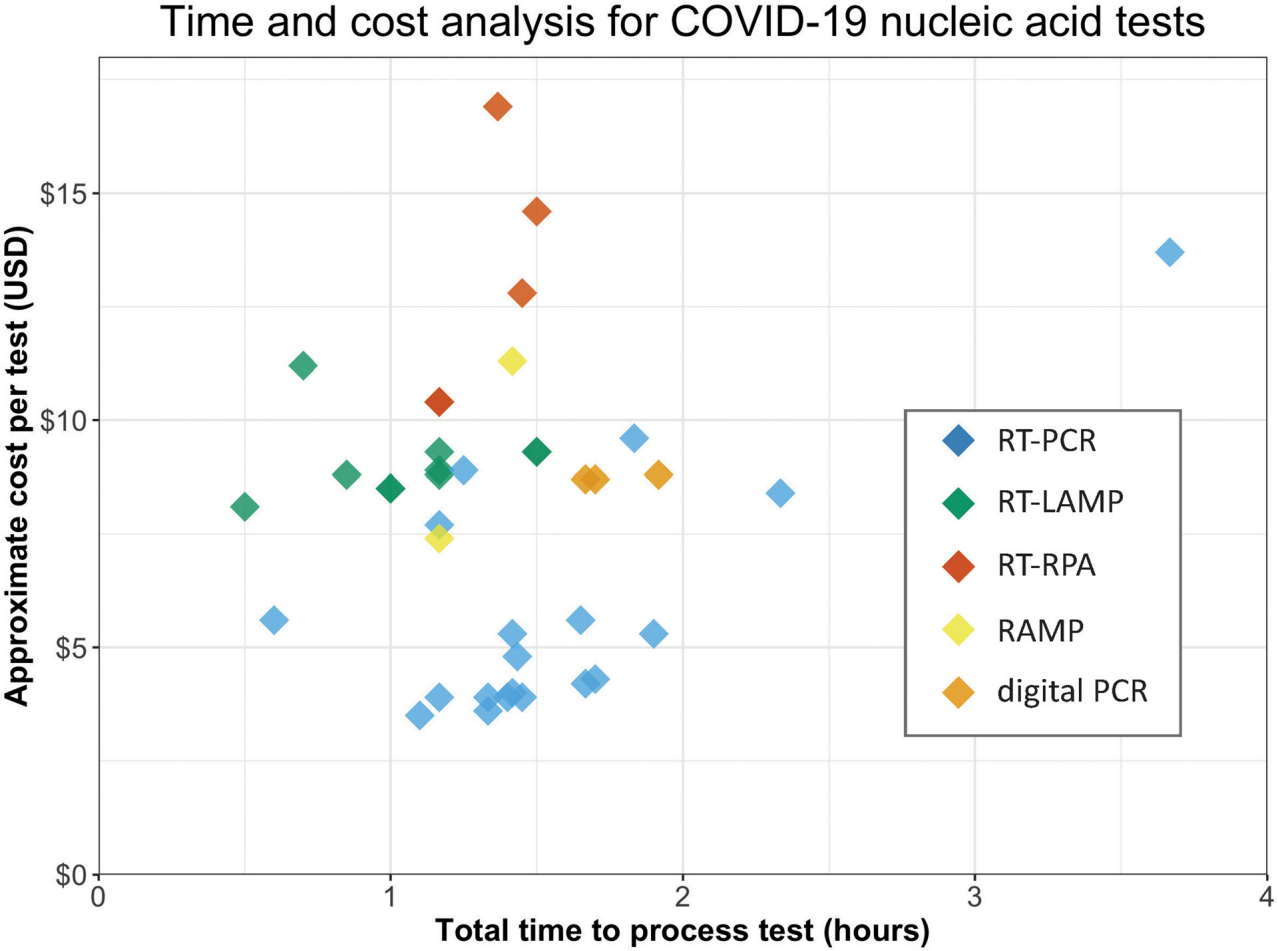
An analysis of the total workflow time and calculated cost (in U.S. dollars) of published COVID-19 nucleic acid tests. Calculated costs are estimated from available online pricing for consumables and do not include labor or equipment. Protocols which required key reagents to be synthesized or created in a laboratory are not included but are likely to be even cheaper than commercially priced reagents. All raw data available in Supplemental Tables S1, S2.

The standard CDC RT-PCR test takes about 3 h to perform and costs ∼$10 per test (Supplemental Table S1). Specialized reagents or equipment can lead to high per-test costs and may limit the number of tests that can be conducted, in some cases resulting in a lag of several days before a patient receives a diagnosis. The variety of approaches presented here span a wide range of costs and processing times, with several published protocols reaching results in less than 1 h (Fig. 3). Some investigators have found homemade solutions that drastically decrease the required reagent cost allowing for tests to be performed for just a few dollars (Supplemental Table S2). Others have proposed completely novel solutions that can cut the testing time to tens of minutes but may still require costly reagents to perform. While widespread testing will necessarily require high-throughput approaches, other tests may offer higher sensitivity for low titer cases or rapid turnaround for point-of-care diagnosis. Recent ingenuity in COVID-19 nucleic-acid testing offers a wide range of solutions and further innovation may be required to maximize testing accuracy while providing a low-cost and fast-turnaround solution.

## SAMPLE LYSIS AND DIRECT ADDITION

**FIGURE 4.**
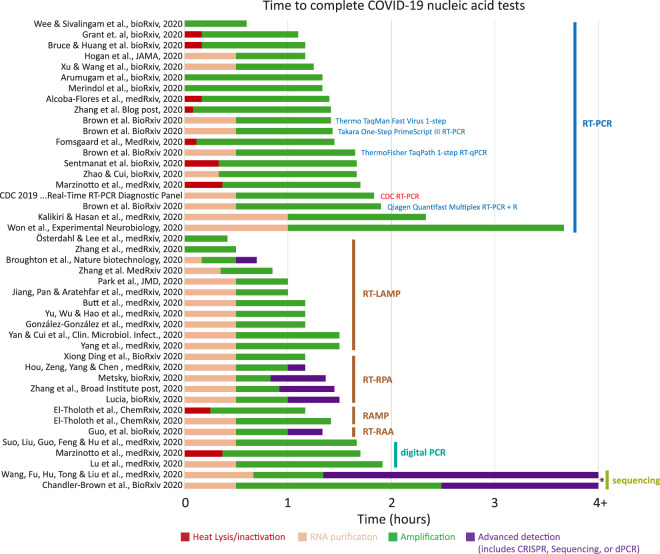
Examination of the total workflow for published COVID-19 testing methods. Each step of the workflow is shown with colored bars. Four example commercial RT-PCR kits are included for reference (blue) and were directly compared within a single publication. The CDC RT-PCR test is shown in red. (*) Sequencing typically takes 4–12 h but can vary significantly depending on library preparation and the platform used, and was not specifically stated in the cited protocols. Raw data available in Supplemental Table S1.

Testing for the presence of SARS-CoV-2 viral RNA typically begins with the collection of a patient swab sample which is stored and transported to a testing facility in viral transport medium (VTM). These samples are lysed and viral RNA is typically purified using either RNA extraction columns or magnetic beads (Fig. 2; Centers for Disease Control and Prevention 2020). One advantage of RNA purification is that the viral RNA present in the more dilute swab sample can be concentrated and eluted in a buffer compatible with RT-PCR. However, in order to decrease reliance on commercial lysis buffers and viral RNA extraction kits and simplify COVID-19 testing, there has been great interest in finding alternative strategies or eliminating RNA purification altogether by adding patient swab samples directly to the RT-PCR reaction. Additionally, eliminating RNA purification can dramatically speed up the overall workflow time per test and may be an ideal solution for streamlining testing times (Fig. 4).

Swab samples must be lysed to release viral RNA into solution for purification and to neutralize the virus for safe handling. Many protocols use commercial reagents for lysis, including DNA/RNA Shield (Zymo Research), Buffer RLT (Qiagen), and MagNA Pure External Lysis Buffer (Roche). However, multiple researchers have recently found that when compared to commercial solutions, homemade solutions containing 4M (Scallan et al. 2020; Sridhar et al. 2020) or 5M (Aitken et al. 2020) guanidinium thiocyanate work equally well to lyse samples and recover viral RNA after purification. However, these solutions contain strong denaturants and are therefore not compatible with addition directly into amplification reactions. Other laboratories have assessed lysis conditions that are compatible with direct addition in order to streamline sample preparation and reduce overall test time. Preliminary studies report that direct-to-test addition of unprocessed swab samples generally allows for SARS-CoV-2 detection but may decrease test sensitivity. Viral RNA stability and compatibility with downstream reactions will be heavily dependent on the buffer used for swab collection and transport. Arumugam and Wong have shown that RNA can be detected from nonreplicative recombinant virus particles (SeraCare AccuPlex*)* in VTM spiked directly into the RT-PCR master mix without an RNA extraction step (Arumugam and Wong 2020). Merindol et al. (2020) compared a few common swab collection buffers for compatibility with direct PCR addition. Swab samples stored in Hank's medium or saline solution and directly added to RT-PCR reactions amplified poorly using either the RealStar SARS-CoV-2 RT-PCR kit (Altona Diagnostics) or the Allplex 2019-nCoV RT-QPCR kit (Seegene) compared to purified RNA from the same swabs. Interestingly, however, viral RNA added directly from swabs stored in water or UTM (Remel) at 4°C showed equivalent RT-PCR amplification to RNA purified from the same swabs. In the presence of RNase inhibitor, viral RNA could be amplified with similar efficiency from swabs stored in water at 4°C for up to 5 d (Merindol et al. 2020).

Many groups are further optimizing direct-to-test addition by heating and/or lysing swab samples prior to testing. In one study, direct addition of swab samples in viral transport media to the Luna Universal Probe One Step RT qPCR master mix (New England Biolabs) accurately identified 92% of 155 COVID-19 cases but reached the detection threshold four cycles later (corresponding to a 16-fold loss in detection of starting material) than a test using RNA extracted from a swab sample using the QIAamp Viral RNA Mini kit (Qiagen) (Bruce et al. 2020). This procedure could correctly identify cases across low, medium, or high SARS-CoV-2 RNA copy loads (as defined by cycles to detection from tests of the same samples after RNA purification). Heating the swab sample at 95°C for 10 min before direct-to-test addition improved detection of low copy load samples (Bruce et al. 2020). Another group reported that directly added samples were detected 3.5 cycles later than RNA isolated using the MagNA Pure kit (Roche), but heating the sample at 95°C for 5 min before direct-to-test addition resulted in detection only one cycle later, with 97.4% accuracy compared to tests using purified RNA (Fomsgaard and Rosenstierne 2020). However, Grant et al. (2020) found the opposite—heating direct-to-test samples in VTM at 95°C for 10 min delayed detection of viral RNA compared to directly added samples not heated prior to amplification. Additionally, they found that direct sample addition in VTM without heating to the TaqMan Fast Virus 1-step Master Mix (Thermo Fisher) RT-qPCR reaction allowed detection 3.77 cycles earlier than the same test performed with RNA purified using the EZ1 Qiagen kit. Overall, their test using a direct, unheated sample had 98.8% diagnostic accuracy when compared to cartridge-based RNA purification and RT-qPCR using the Panther Fusion system (Grant et al. 2020). Intermediate inactivation temperatures seem to perform worse than high heat or no heating at all. One group reported that swab sample heat treatment at 75°C for 10 min prior to direct-to-test addition delayed detection by 6.1 cycles (Alcoba-Florez et al. 2020). Multiple groups have reported contradictory findings on the advantages of heating samples before direct addition into RT-PCR mixes. RNases present in the nasal swab are likely active even at high temperatures and thus RNA degradation may be particularly sensitive to the temperature and buffer conditions of inactivation.

Slightly more complex approaches use lysis buffers to aid RNA recovery and improve RT-qPCR test sensitivity. In one report, positive patient swab samples diluted 1:1 into Quick Extract DNA extraction solution (a buffer containing detergents and proteinase K), heat inactivated and directly added to the RT-PCR reaction mix were detected at the same amplification cycle as, or even slightly before, samples processed with the QIAmp Viral RNA Miniprep kit (Qiagen) (Ladha et al. 2020). Another group reported that swab samples added to Quick Extract DNA extraction solution were detected with equal sensitivity to column-purified RNA (Sentmanat et al. 2020). Finally, another study reported that direct addition of swab samples treated 15 min with proteinase K yielded sensitivity comparable to the use of RNA isolated with the automated ELITe InGenius Sp200 system (ELITech Group) (Marzinotto et al. 2020).

The discrepancy between the sensitivities of direct-to-test addition procedures may be due to differences in protocols and kits used for the RT-qPCR test and isolation of control RNA, types of lysis buffers, heating parameters, and varying viral RNA loads in the swab samples of each study. Despite these discrepancies, it appears that direct-to-test addition of a small volume of swab sample treated with lysis buffer or Proteinase K allows for robust SARS-CoV-2 RNA detection. Direct-to-test addition of patient swab samples may prove useful in settings where there is a lack of RNA purification reagents or time constraints that render laborious RNA isolations infeasible. Further work is required to ascertain optimal swab sample lysis, heating and storage conditions prior to direct-to test addition, as well as whether direct-to-test addition could be used in tests other than RT-qPCR.

## RNA PURIFICATION

As uncovered by multiple groups, eliminating RNA isolation prior to RT-PCR altogether may be possible. However, a dedicated RNA isolation step may improve detection sensitivity or be necessary to remove incompatible sample buffers prior to amplification for some protocols. However, column-based kits used to purify RNA from the patient swab sample can also occasionally lead to unintentional carryover of ethanol or retention of some RNA, which can be kit-specific. In our laboratory, we have found that the RNeasy Mini Kit (Qiagen) leads to an approximately eightfold (3 *C*_t_) loss of synthesized SARS-CoV-2 viral *N* gene RNA after column purification. We found similar results with inactivated positive patient swab samples; *C*_t_ values were consistently lower when RNA was purified via isopropanol precipitation or using the Direct-zol RNA Miniprep Plus kit (Zymo Research) when compared to the RNeasy Mini Kit (Qiagen). However, we have not compared directly with the CDC recommended Qiagen QIAmp Viral RNA kit (C Dugast-Darzacq, T Graham, GM Dailey, et al., unpubl.). Several recent papers have investigated alternative methods for RNA purification, including unique approaches as well as traditional laboratory techniques. Zhao et al. (2020) present a synthesis protocol for magnetic nanoparticles that can combine sample lysis and RNA binding in a single step. The polyamino ester with carboxyl groups-coated magnetic nanoparticles (pcMNPs) are also directly compatible with the RT-PCR reaction, greatly streamlining the protocol from lysis through RNA purification, and the pcMNPs can be synthesized on-site. Kalikiri et al. (2020) find that AmpureXP beads (Beckman Coulter) yield equal sensitivity to the NucliSENS easyMAG automated extraction platform (bioMérieux). Other commonly used laboratory reagents for RNA purification include TRIzol, which includes guandinium thiocyanate and phenol-chloroform to extract RNA from cellular samples. Won and coworkers describe a complete workflow for COVID-19 testing which includes TRIzol extraction and isopropanol precipitation of the RNA from swab samples. The authors found no difference between TRIzol and the approved Qiagen QIAamp Viral RNA Mini Kit in RNA extraction efficiency from Lentivirus-infected HEK293 cells, but was not directly compared using SARS-CoV-2 RNA (Won et al. 2020). In our laboratory, isopropanol precipitation of synthesized SARS-CoV-2 viral *N* gene RNA resulted in almost no loss of RNA, with or without the presence of additional human RNA as a carrier (C Dugast-Darzacq, T Graham, GM Dailey, et al., unpubl.). Standard laboratory RNA purification methods offer an attractive alternative to commercial kits, as they generally use inexpensive, abundant materials. For clinical testing, however, these solutions may be difficult to scale to high-throughput pipelines and may require special handling of hazardous materials. It may be useful to assess where RNA extraction can be eliminated while maintaining the necessary sensitivity and accuracy of testing. If eliminating RNA purification is not possible, however, these procedures could be useful as cheap, homemade solutions for small-scale testing operations.

## RT-PCR

**FIGURE 5.**
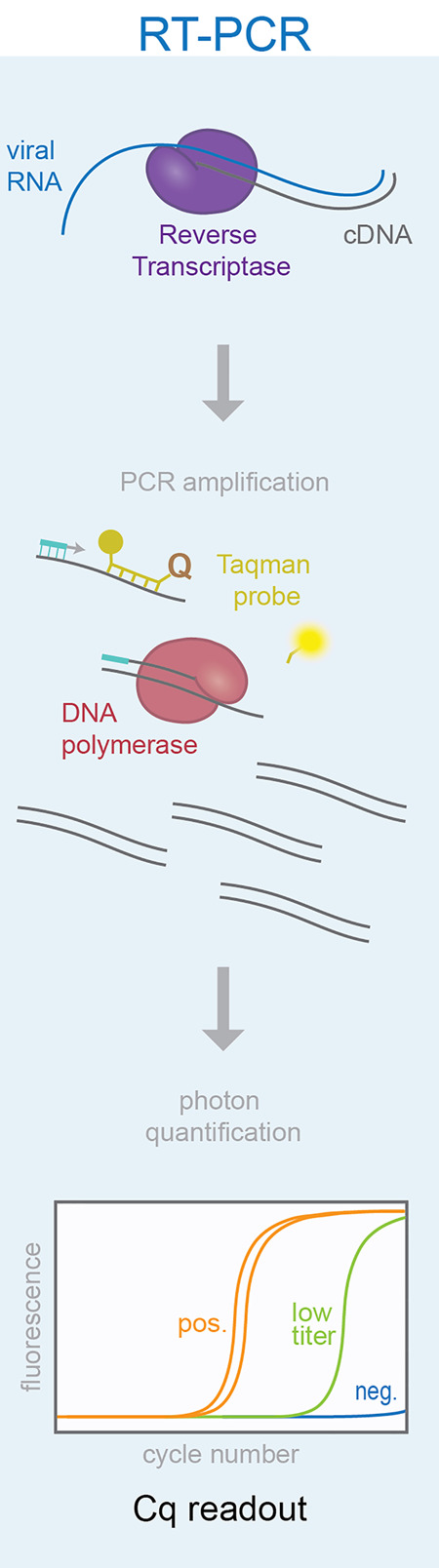
Molecular overview of the RT-PCR reaction. Taqman probes are used to visualize increased fluorescence during each cycle of amplification. Amplification is quantified by *C*_q_ readout and a threshold is set for positive detection of the target amplicon.

RT-PCR master mixes use a mixture of reverse transcriptase enzymes, such as the thermostable MMLV RT, and a DNA polymerase, like Taq. Primers that anneal specifically to the SARS-COV-2 viral genome are included to prime amplification. The U.S. CDC protocol utilizes primers that target the viral *N* gene, while the China CDC uses primers matching both the *N* gene and the *ORF1ab* region, and Charité Germany primers target the *RdRp* and *E* genes (for review, see Udugama et al. 2020). For detection of amplification, qPCR can be performed using intercalating dyes like SybrGreen. Because these dyes are nonspecific for DNA products, any amplification (specific or not) will lead to an increased fluorescent readout. Higher sequence specificity in the detection of amplicons can be achieved using Taqman probes. These short oligonucleotides contain a 5′ fluorophore and 3′ quencher and anneal to sequences within the DNA template. Taq polymerase degrades the annealed probe by its 5′ to 3′ exonuclease activity and cleaves off the fluorophore, thereby releasing it from being quenched. This fluorescence is proportional to the number of amplified product molecules, is sequence-specific for the correct amplified product, and can be measured in real-time on a qPCR machine (Fig. 5).

**FIGURE 6.**
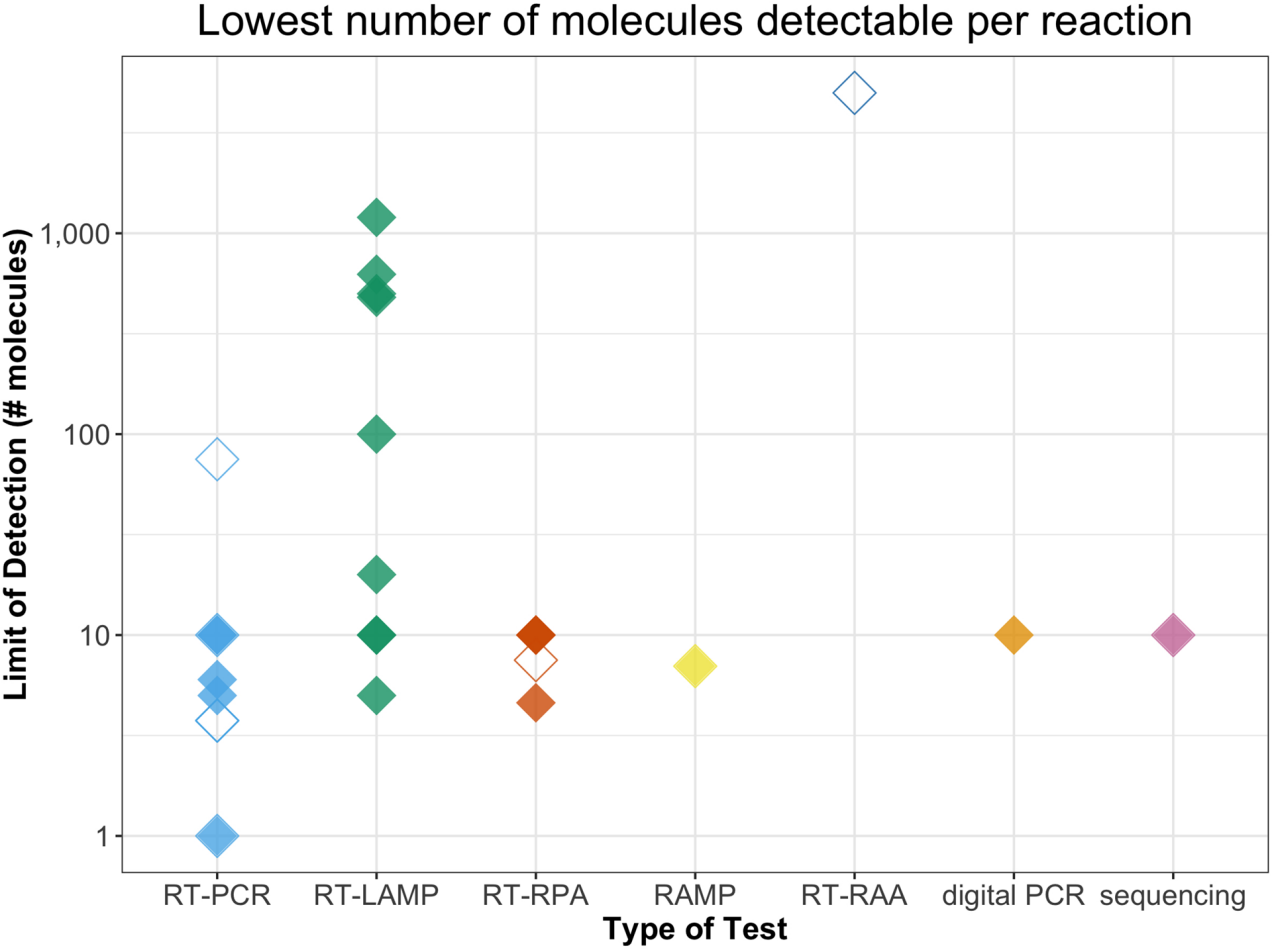
The limit of detection for published tests equivalent to the fewest number of molecules accurately assayed in a single reaction. For some spike-in controls, authors used viral DNA, plasmid DNA, or a pseudovirus instead of viral RNA (shown as open diamonds), which may have a different amplification efficiency than SARS-CoV-2 RNA and thus alter their calculated limit of detection. Raw data available in Supplemental Table S1.

RT-PCR has been deemed the “gold standard” for COVID-19 diagnosis because it has shown to be very sensitive for accurately detecting viral genomes present, down to just one molecule of RNA (Fig. 6). Multiple commercial master mixes exist that enable sensitive one-step RT-PCR. The original CDC protocol approved four commercial master mixes for the RT-PCR test from Quantabio, Promega, and ThermoFisher (Centers for Disease Control and Prevention 2020). However, published RT-PCR protocols have also successfully used one-step RT-PCR master mixes from a variety of companies including NEB, Applied Biosciences, Qiagen, Roche, Takara, and others, and a growing list of approved alternative commercial reagents can be found at the FDA EUA website (Alcoba-Florez et al. 2020; Chandler-Brown et al. 2020; Food and Drug Administration 2020; Kalikiri et al. 2020; Marzinotto et al. 2020; Merindol et al. 2020; Won et al. 2020; Xu et al. 2020; Zhao et al. 2020). Many commercial master mixes seem to function well in the detection of SARS-CoV-2, although a detailed side-by-side comparison of the numerous commercial reagents is lacking. Brown et al. (2020) compared four popular one-step RT-PCR kits (Takara One Step PrimeScript III kit, Qiagen Quantifast Multiplex RT-PCR + R Master Mix, ThermoFisher TaqPath 1-step RT-qPCR Master Mix, and the Thermo Fisher Taqman Fast Virus 1-step Master Mix) on 74 patient nose and/or throat swabs. Comparison of the four master mixes showed that three out of the four mixes performed optimally with the N2 primers for SARS-CoV-2 detection—the Takara, Qiagen, and TaqPath. The best, however, seemed to be the Takara master mix, which was able to detect just a single viral genome copy using the N1 primers. Consistent with the Takara mix being the most sensitive, none of their patient samples that tested negative with the Takara mix tested positive with the Qiagen kit, whereas the reverse did not hold (Brown et al. 2020).

Additionally, in order to decrease reliance on a particular company to generate master mix reagents for testing, which in the course of the pandemic could experience supply chain disruptions or delays, at least one laboratory has developed a completely homemade, open-source master mix. Bhadra et al. have developed master mixes using the evolved reverse transcriptase/DNA polymerase RTX that are compatible both with either dye-based or TaqMan qPCR. The RTX enzyme can be expressed in *E. coli* and purified using Ni-NTA agarose and heparin columns, and master mix buffers can be made easily and cheaply in a laboratory. The authors demonstrated detection of as few as 100 molecules of in vitro transcribed SARS-CoV-2 N gene RNA, using either RTX enzyme alone in a dye-based reaction or a mixture of RTX and Taq in a TaqMan reaction. TaqMan reactions with RTX and Taq showed Cq values comparable to the commercial TaqPath kit (Bhadra et al. 2020). Future studies should assess homemade master mixes using patient samples, to provide inexpensive, open-source options for testing. While a variety of commercial and laboratory options exist for RT-PCR master mixes, active enzymes typically require careful refrigeration for storage and transport. Xu et al. (2020) have demonstrated that the Takara RT-PCR mix maintains its activity after being freeze-dried and stored at room temperature for 28 d. Further innovation in homemade or room-temperature stable reagents may improve testing capabilities in remote locations or at the point-of-care.

## ISOTHERMAL AMPLIFICATION

**FIGURE 7.**
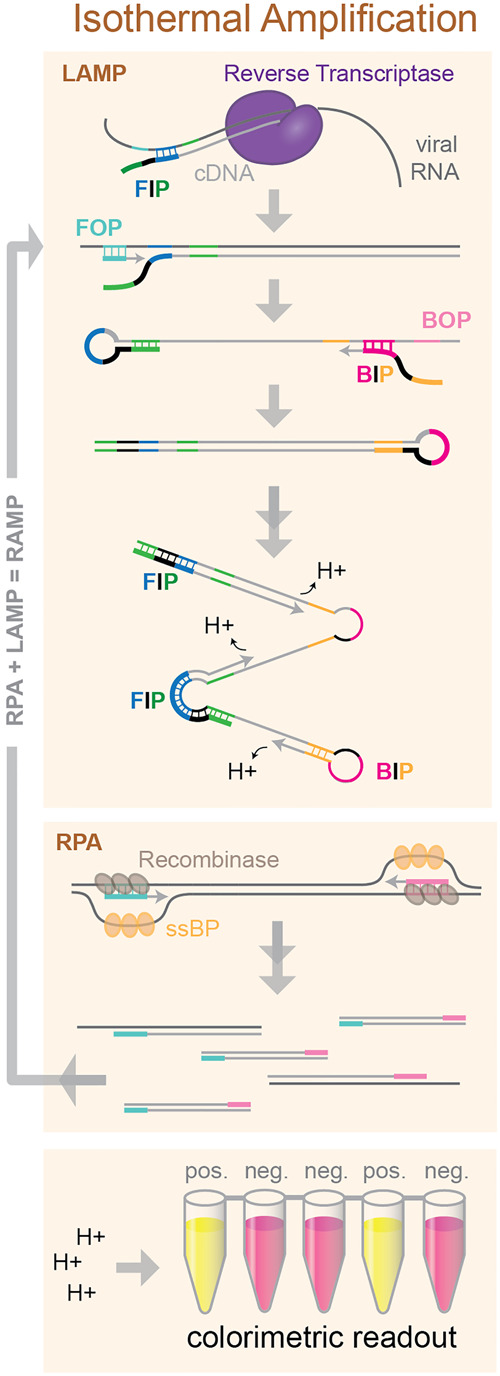
Molecular overview of isothermal amplification techniques. LAMP uses specially designed nested primers with complementary regions that form hairpins to permit priming of subsequent rounds of amplification. RPA uses recombinase-catalyzed strand invasion to prime amplification. Colorimetric pH indicators can be used to detect hydrogen ion release during dNTP incorporation.

A promising alternative to RT-PCR is isothermal amplification, which does not require thermocycling. Two isothermal techniques used for rapid and sensitive diagnostics are loop-mediated isothermal amplification (LAMP) and recombinase polymerase amplification (RPA) (Fig. 7). LAMP uses a strand-displacing DNA polymerase together with four specially designed primers containing regions of complementarity to six target sequences. The 3′ end of the forward inner primer (FIP) primes synthesis of an initial DNA strand, which is subsequently displaced by synthesis primed by the forward outer primer (FOP). A reverse-complementary sequence in the 5′ end of the FIP anneals with a downstream sequence in the displaced ssDNA strand, forming a loop. The same process repeats with the backward inner and outer primers (BIP and BOP) at the opposite end of the amplicon. Repeated rounds of priming and strand extension generate a mixture of stem–loop and “cauliflower” structured products. Because LAMP includes primers that anneal to six unique target regions, it is highly sequence specific (Notomi et al. 2000). Release of hydrogen ions upon incorporation of dNTPs into the nascent DNA chain can be detected using colorimetric pH indicator dyes (Tanner et al. 2015). RT-LAMP has been validated for detection of a multitude of RNA viruses including influenza, Zika, Ebola, and MERS (for review, see Wong et al. 2018).

A slightly more recent addition to the isothermal amplification toolkit is RPA. RPA uses a recombinase to catalyze strand invasion of a primer into dsDNA. Single-stranded binding proteins are included to stabilize the open duplex structure and a strand-displacing DNA polymerase extends the primer (Piepenburg et al. 2006). Some groups have demonstrated very high sensitivity and specificity of target amplification by combining RPA and LAMP into a two-stage amplification protocol, termed RAMP. The outer LAMP primers can be used for RPA amplification and then combined with the additional LAMP primers for further amplification in a single tube or microfluidic device. The combined RAMP approach exhibits the extremely high specificity of LAMP, together with enhanced sensitivity from dual amplification, and a higher tolerance to inhibitors (Song et al. 2017). Song et al. (2017) demonstrated the huge potential of the RAMP approach for diagnostics by multiplexing 16 pathogenic targets including HIV-1 and multiple strains of HPV ZIKV. These isothermal methods are relatively fast and can be read out colorimetrically, with a lateral-flow stick, or even with nanoparticle-based biosensors (Zhu et al. 2020), making them easy to use at home or at remote points of care.

Several groups have now developed novel isothermal protocols for detection of SARS-COV-2 RNA. Lu et al. (2020b) tested their LAMP-based detection method with spiked-in SARS-COV-2 RNA and were able to detect a colorimetric change indicating a positive result after just 40 min of amplification, with sensitivity down to 30 viral RNA copies per reaction. They and others have demonstrated that LAMP detection of SARS-CoV-2 is specific by showing no cross-reactivity to other respiratory pathogens including human coronavirus strains HCoV-OC43 and HCoV-229E (Lu et al. 2020b; Park et al. 2020). Zhang et al. have shown that their LAMP strategy gives results that match the RT-PCR standard test in COVID-19 positive patient samples, reporting 100% sensitivity and specificity. They also find that the LAMP protocol may be compatible with cell lysates, potentially eliminating the need for RNA purification from patient samples (Zhang et al. 2020a). Using 130 samples, Yan et al. were able to directly compare RT-PCR with RT-LAMP. The LAMP assay gave identical clinical diagnoses to the RT-PCR test, with similar sensitivity, and it was faster and easier to read out (Yan et al. 2020). Others have reported similar success, with LAMP amplification yielding 90%–100% sensitivity and 95%–99% specificity in patient samples with improved accuracy for amplification of multiple gene targets (Jiang et al. 2020; Mehmood Butt et al. 2020; Yang et al. 2020; Yu et al. 2020). A smaller cohort study found that their RT-LAMP test had a sensitivity of 80%, compared to consecutive RT-PCR swabs, which could be adequate clinically, they suggest, if repeated testing were used (Österdahl et al. 2020). By combining two common isothermal techniques, LAMP and RPA, into a single-tube RAMP reaction, El-Tholoth and colleagues were able to improve detection 100-fold over RT-PCR in mimic patient samples, providing an early proof-of-concept for an extremely sensitive method that can detect down to just a few viral RNA copies, but that to date has not yet been tested on patient samples (El-Tholoth et al. 2020). From these early demonstrations, under optimized conditions isothermal amplification techniques can provide equal sensitivity and specificity to the RT-PCR test for SARS-CoV-2 detection. These methods allow for faster amplification, less specialized equipment, and easy readout. LAMP methods also benefit from the ability to multiplex targets in a single reaction and can be combined with other isothermal methods, like RPA in the RAMP technique, to increase test accuracy even more. These techniques may be particularly useful for rapid, point-of-care diagnoses or for remote clinical testing without the need for laboratory equipment.

## CRISPR-BASED DETECTION

**FIGURE 8.**
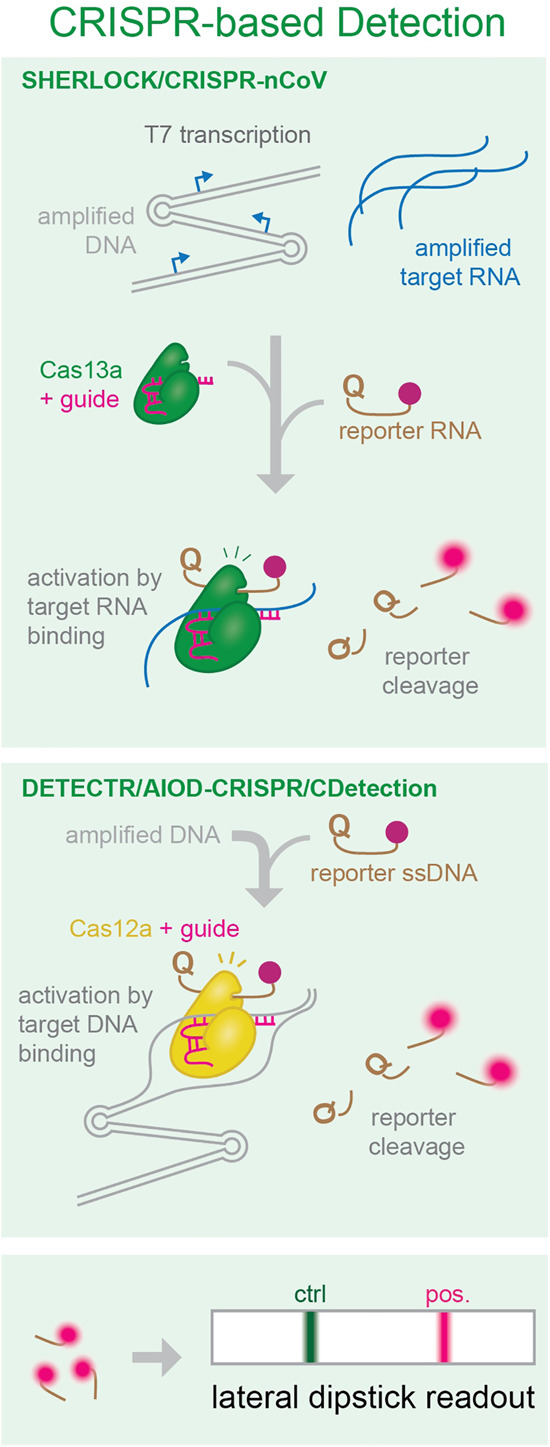
Molecular overview of CRISPR detection of amplified products. Binding to specific target sequences in amplified RNA or DNA activates Cas nucleases, which cleave reporter molecules. Reporter cleavage can then be assayed using a lateral dipstick.

A unique group of Cas nucleases, including Cas12 and Cas13 were recently discovered to have promiscuous DNA or RNA cleavage activities (Gootenberg et al. 2017; Chen et al. 2018; Li et al. 2018), which have been exploited for nucleic acid detection. Multiple assays combining isothermal amplification and CRISPR have recently emerged as diagnostic tools for rapid detection of SARS-CoV-2 viral RNA (Fig. 8).

Cas13a is a nonspecific RNase that remains inactive until it binds its programmed RNA target. It has been harnessed for sensitive DNA or RNA detection in a method termed SHERLOCK (Gootenberg et al. 2017) In SHERLOCK, the target RNA is first amplified by a combination of RT-RPA and T7 transcription. The amplified product RNA activates Cas13a, which in turn cleaves a reporter RNA, liberating a fluorescent dye from a quencher. This method consistently detects synthetic SARS-CoV-2 viral RNA in the range between 10 and 100 copies per µL of input, only requires a lateral flow dipstick for visual readout of the detection result, and can be completed in 40 min (Hou et al. 2020) or 57 min (Metsky et al. 2020; Zhang et al. 2020b) after the RNA extraction step. SHERLOCK (termed “CRISPR-nCoV” in Hou et al. 2020) also demonstrated its diagnostic potential by detecting SARS-CoV-2 RNA with 100% sensitivity in 52 patient samples (Hou et al. 2020).

Cas12 is another member of the CRISPR-Cas effector family. It is an RNA-guided DNase that indiscriminately cleaves ssDNA upon binding its target sequence. In a method termed “DETECTR,” Cas12a ssDNase activation is combined with isothermal amplification to achieve sensitive and specific DNA detection (Chen et al. 2018). Multiple groups have recently used DETECTR for SARS-CoV-2 RNA detection. Viral RNA is first converted to DNA and isothermally amplified. Specific target sequences in the amplified DNA activate Cas12a, which in turn cleaves a ssDNA reporter to unquench a fluorophore. Using RT-RPA for amplification in DETECTR, Lucia et al. (2020) detected 10 copies of SARS-CoV-2 RNA per µL of input within 60 min (after RNA sample preparation). Ding et al. improved the protocol by combining RT-RPA and CRISPR-based detection in a one-pot reaction and incubating at a single temperature. This “All-In-One Dual CRISPR-Cas12a” (AIOD-CRISPR) assay detected as few as 4.6 copies of SARS-CoV-2 RNA per µL of input in 40 min (Ding et al. 2020). Similarly, Guo et al. developed another single-tube and constant temperature protocol (“CDetection”), in which they used recombinase-aided amplification (RAA) instead of RPA for nucleic acid amplification. They showed that Cas12b behaves similarly to Cas12a for ssDNA reporter cleavage and can achieve a detection limit of five copies/µL in 40–60 min (Guo et al. 2020). Moreover, Broughton et al. used LAMP instead of RPA in DETECTR and further reduced the testing time for SARS-CoV-2 RNA to 30–32 min while maintaining a low detection limit (10 copies/µL) (Broughton et al. 2020).

In combination with fast isothermal amplification, CRISPR-based techniques can harness highly specific nucleases to achieve fast read-outs and sensitivity down to a few viral RNA copies. CRISPR detection can be coupled to lateral flow readouts, which are an attractive option for easy, at-home testing scenarios.

## SEQUENCING FOR DIAGNOSIS

Sequencing-based detection methods provide the benefit of collecting base-pair-level information of patient strains, which allows for viral mutation tracing but comes at the cost of expensive sequencing platforms and lengthy sample processing times. However, several laboratories have investigated high-throughput approaches or portable, fast sequencing to use this technology as a diagnostic tool for COVID-19. Nanopore target sequencing (NTS) is an attractive option for clinical testing because it is fast, highly portable, and sensitive. Wang et al. have developed an NTS approach targeting 11 viral regions that is able to detect as few as 10 viral copies/mL with 1 h of sequencing. By relying on a sequencing-based approach, this group also demonstrated that viral genome mutations can be identified within the target regions, and that an additional panel of targets against common respiratory viruses can be included to detect co-infection (Wang et al. 2020). Additionally, commercial sequencing approaches have also been adapted for high-throughput SARS-CoV-2 testing. BillionToOne Inc. seeks to use the extensive national infrastructure for Sanger sequencing, which they propose could “unlock more than 1,000,000 tests per day in the US” (Chandler-Brown et al. 2020). BillionToOne uses a one-step RT-PCR mix to amplify viral RNA directly from swab samples, which are collected in viral transport medium rather than a custom lysis buffer. Sanger sequencing then proceeds with inclusion of a synthetic, shortened SARS-CoV-2 sequence as a spike-in control allowing for careful quantitation of viral abundance down to ∼10 genomic equivalents (Chandler-Brown et al. 2020). While traditional sequencing approaches typically require substantial cost and specialization, repurposed portable or quantitative sequencing approaches may offer extremely accurate high-throughput diagnostics during the pandemic.

## OTHER NATs (“THINKING OUTSIDE THE BOX”)

Beyond the approaches described above, ingenious methods are being developed for widespread, at-home, or point-of-care COVID-19 diagnostics. Most isothermal amplification steps require incubation at elevated temperatures around 60°C. To facilitate isothermal amplification at remote testing facilities, González-González et al. (2020) developed a 3D-printed water circulator that can act as a heat block for LAMP amplification and have demonstrated the ability to detect as few as 62 viral RNA molecules after 1 h of incubation. To make RT-PCR more accessible for remote testing, Wee et al. (2020) have demonstrated a rapid, extraction-free PCR protocol that can detect six SARS-CoV-2 RNA copies using a portable thermocycler.

While samples collected from patients with symptoms or who have been hospitalized seem to present relatively high viral titers that are likely to be easier to detect (Wölfel et al. 2020), testing of asymptomatic patients or testing prior to quarantine release may require extremely sensitive tests. While specialized reagents and equipment are required, digital and digital-droplet PCR may allow for even more sensitive testing than RT-PCR. Lu et al. (2020a) report 96.3% accuracy for testing of clinical samples using digital PCR and were able to detect virus in four patient samples that were deemed negative by RT-PCR. Furthermore, digital droplet methods have been shown to be capable of detecting down to 0.4 viral RNA copies/μL in patient samples (Suo et al. 2020). Because digital PCR allows for more careful quantitation of viral RNA copy number over the course of the disease, this highly sensitive test may also be useful to evaluate treatment progress or assess patient release after quarantine.

Particularly as testing becomes more widespread, testing of the general population and asymptomatic individuals may lead to a large number of negative samples and a huge increase in the demand of testing supplies. In an innovative effort to further conserve resources using existing testing methodologies, some groups have investigated pooling many patient samples to decrease the number of tests required for larger populations. Proposed pooling approaches can be adaptive, where samples are first pooled and tested and positive pools are retested individually. This is a relatively simple solution which decreases overall testing resources used but may introduce several disadvantages, including longer wait times for results since positive samples must be iteratively tested, and a slight loss in sensitivity from diluting positive patient samples with negative ones. Multiple groups have modeled patient pooling and proposed algorithms that optimize positive sample detection and testing efficiency (Noriega and Samore 2020; Sinnott-Armstrong et al. 2020). Some simple approaches like pooling the rows and columns of a 96-well plate during testing can increase efficiency four- to eightfold for low prevalence populations (Sinnott-Armstrong et al. 2020). Random pooling has also been shown to be useful for estimating disease prevalence and transmission within a local area (Hogan et al. 2020). More complicated pooling assignments and nonadaptive approaches have been proposed and may significantly increase efficiency and allow for single-iteration pooled testing, but may be more difficult to implement with common clinical workflows and robotic pipetting (Täufer 2020). The solution to widespread testing is likely to require an adaptive, multipronged approach. While pooling of samples may not be the most appropriate solution for very sensitive testing, pooling may drastically improve our ability to screen large populations while conserving limited testing resources.

Lastly, it is important to consider that enzyme-based tests are not always feasible in resource-limited scenarios. One potential alternative lies in the use of DNA “nanoswitch”-based tests that have been developed for Zika virus detection. Based on a DNA origami design, these nanoswitch DNA oligomers bind viral RNA to undergo a conformational change that can be visualized on an agarose gel. DNA nanoswitches targeting different species of RNA viruses can also be combined in one test, allowing for the detection of co-infections. Unfortunately, the Zika nanoswitch test requires ∼5.2 × 10^5^ Zika RNA genomes per test for reliable detection and is thus far less sensitive than RT-PCR (Zhou et al. 2020). Due to being a gel-based method, the throughput of DNA nanoswitch tests is also severely limited. To avoid low-throughput gel detection, the Godin group developed a nanopore sensor capable of detecting these conformational changes and could detect as few as ∼500 target molecules (Beamish et al. 2019). Further innovation in DNA nanoswitch detection of SARS-CoV-2 and developments in generating fluorescent or colorimetric outputs could significantly improve the test's throughput and facilitate its use for COVID-19 detection in low-resource areas.

## OUTLOOK

In response to the tremendous global toll of COVID-19, researchers have rapidly mobilized to investigate solutions for testing, diagnosis, and treatment. Preprint and published articles from the past several months describe a variety of options for rapid, affordable, sensitive, and high-throughput nucleic acid testing, which is currently the most reliable approach for early detection of SARS-CoV-2. To address the dire need for increased testing, researchers across disciplines have quickly compared widely available commercial products, proposed repurposing existing reagents and infrastructure, and created novel laboratory solutions to optimize the COVID-19 testing pipeline. Academic researchers at a few institutions around the world have published detailed blueprints for establishing local pop-up testing centers, and others are likely to follow (Aitken et al. 2020; Innovative Genomics Institute SARS-CoV-2 Testing Consortium and Doudna 2020; Sridhar et al. 2020). A combination of testing approaches may be the most efficient way to fill the current gaps in testing. We are hopeful that the explosion of creative and multifaceted approaches to COVID-19 nucleic-acid testing will continue to seed solutions as society addresses the COVID-19 pandemic.

## GLOSSARY

RT-PCR—reverse transcription polymerase chain reaction; amplification of RNA in a one-step reaction containing a reverse transcriptase enzyme, DNA polymerase, and a specific primer complementary to a target region.

LAMP—loop-mediated isothermal amplification that uses four primers that recognize six complementary regions within the target. Amplification occurs from a strand-displacing polymerase and elongation of primers with self-complimentary regions for hairpins that prime further rounds of amplification forming large “cauliflower” structures of amplified products.

RPA—rapid amplification; recombinase polymerase amplification uses a recombinase-primer complex which finds matches in the DNA or RNA template and enables strand exchange to form an open complex. Single-stranded binding proteins stabilize the open duplex and strand-displacing DNA polymerase amplifies the template.

RAMP—a two-stage isothermal amplification technique combining a primary RPA reaction using the outside LAMP primers with a secondary LAMP reaction including self-complimentary internal primers.

*C*_t_ or *C*_q_ value—in qPCR, the amplification cycle where the fluorescence curve exhibits the greatest curvature and exceeds the background fluorescence threshold.

Sensitivity (Baratloo et al. 2015)—the ability of a test to detect a true positive = $\frac{\mathrm{true}\;\mathrm{positive}}{\mathrm{true}\;\mathrm{positive}+\mathrm{false}\;\mathrm{negative}}\;*\;100$.

Specificity (Baratloo et al. 2015)—the ability of a test to detect a true negative = $\frac{\mathrm{true}\;\mathrm{negative}}{\mathrm{false}\;\mathrm{positive}+\mathrm{true}\;\mathrm{negative}}\;*\;100$.

Accuracy (Baratloo et al. 2015)—the ability of a test to differentiate true positive and negative results correctly from the total tests = $\frac{\mathrm{true}\;\mathrm{positive}+\mathrm{true}\;\mathrm{negative}}{\mathrm{true}\;\mathrm{positive}+\mathrm{false}\;\mathrm{positive}+\mathrm{true}\;\mathrm{negative}+\mathrm{false}\;\mathrm{negative}}\;*\;100$.

## SUPPLEMENTAL MATERIAL

Supplemental material is available for this article.

## Supplementary Material

Supplemental Material
