# Supplementary material for: Overcoming the bottleneck to widespread testing: a rapid review of nucleic acid testing approaches for COVID-19 detection
Source: RNA. 2020 Jul;26(7):771–83. doi: 10.1261/rna.076232.120 (PMC7297120; doi:10.1261/rna.076232.120)
Supplement: Supplemental Material [file supp_26_7_771__index.html]

Overcoming the bottleneck to widespread testing: a rapid review of nucleic acid testing approaches for COVID-19 detection — Supplemental Material 

# Overcoming the bottleneck to widespread testing: a rapid review of nucleic acid testing approaches for COVID-19 detection

## Supplemental Material

- Supplemental\_Table\_Legends.docx
- Supplemental\_Table\_S1.xlsx
- Supplemental\_Table\_S2.xlsx
