# Supplementary material for: Overcoming the bottleneck to widespread testing: a rapid review of nucleic acid testing approaches for COVID-19 detection
Source: RNA. 2020 Jul;26(7):771–83. doi: 10.1261/rna.076232.120 (PMC7297120; doi:10.1261/rna.076232.120)
Supplement: Supplemental Material [file supp_076232.120_Supplemental_Table_Legends.docx]

**Supplemental Information**

**Table S1**. The approach, accuracy, sensitivity, speed, throughput, and total cost of each published COVID-19 test that is discussed in the current review. “Time to complete the test” is equal to “Time for RNA preparation” + “Time for amplification and detection” and is used in plots of **Figure 3** and **Figure 4**. “Approximate consumable cost per test (USD)” is calculated in **Table S2** and is plotted in **Figure 3**. For CRISPR methods that list costs for both lateral flow and fluorescence detection, only the fluorescence cost is plotted to more closely compare with other CRISPR protocols that use fluorescent readout. “Standardized LoD (minimum detectable viral RNA copies)” is plotted in **Figure 6**.

**Table S2**. Costs, amounts, and vendors of required reagents for representative COVID-19 tests. Costs include consumables but do not include labor or purchase cost of machinery required. Prices are quoted directly from publicly available vendor websites, current as of April 26, 2020.
